# Development of long-term oyster explant cultures enable sustained tissue-specific activity and reveal novel cellular behaviours

**DOI:** 10.1016/j.bbrep.2026.102775

**Published:** 2026-09-07

**Authors:** Kallen R. Sullivan, Pieter C. Steketee, Scott Maxwell, Liam J. Morrison, Tim Regan, Tim P. Bean

**Affiliations:** a The Roslin Institute, University of Edinburgh, EH25 9RG, UK; b The Royal (Dick) School of Veterinary Studies, University of Edinburgh, EH25 9RG, UK

**Keywords:** *Ostrea edulis*, Explant culture, Long-term tissue culture, Cell culture, Marine invertebrate, Histology

## Abstract

Molluscs are ecologically and economically important, yet progress in understanding their cellular biology has been limited by the lack of reliable cell culture systems. Here, we establish reproducible long-term *in vitro* and *ex vivo* cultures from multiple tissues of the European flat oyster, *Ostrea edulis*, including heart, visceral mass, mantle, and gill. Explanted tissues remained intact and exhibited tissue-specific function for over two months, with whole hearts maintaining rhythmic contractions for up to nine months. Cultures revealed cellular plasticity, including haemocyte granule turnover, auricular epithelial remodelling with novel ciliation, and the emergence of multicellular microtissues. Previously understudied cell types, including visceral mass adipocytes and mantle myocytes, were maintained in culture, expanding the cellular repertoire accessible *in vitro*. These systems provide a tractable platform for future molluscan studies, while establishing a foundation for comparative invertebrate cell biology and the future development of immortalised molluscan cell lines.

## Highlights

• Reproducible long-term cultures were established from multiple oyster tissues.
• Explanted oyster tissues retained tissue-specific function for over two months.
• Whole oyster hearts maintained rhythmic contractions for up to nine months.
• Cultures revealed cellular plasticity, remodelling, and microtissue formation.
• Understudied oyster cell types were maintained and accessible *in vitro*.

## 1 Introduction

Cell culture systems provide a critical bridge between organism-level biology and the underlying cellular biology. Experimental *in vitro* systems enable detailed exploration of cell physiology, behaviour and disease modelling, thereby progressing our biological understanding. For molluscs, whose ecological functions and aquaculture value are globally significant, the absence of stable, reproducible culture systems has hindered progress in studies ranging from immunity and stress physiology to biomineralization and disease management.

Molluscs are among the most diverse and ecologically significant animal phyla, comprising more than 86,000 described species and likely exceeding 200,000 across marine, freshwater, and terrestrial ecosystems [1]. They occupy critical ecological roles while also underpinning global aquaculture, fisheries, and biotechnological industries [2]. Oysters, in particular, highlight this importance: global aquaculture production is approximately 5.5 million tonnes, and they provide ecosystem services such as water filtration, sediment stabilization, and reef formation that enhance coastal biodiversity [3,4]. Beyond industry and ecology, molluscs are established models in neurobiology, biomineralization, and ecotoxicology [5]. Yet, despite this breadth of significance, fundamental insights into their cellular biology remain limited, constrained by persistent challenges in establishing reliable primary cell cultures [6].

Attempts to develop molluscan cell cultures date back several decades [6,7]. Haemocytes, the circulating immune cells of molluscs, are the most frequently studied cell type because they are readily accessible and play a central role in immune response and wound healing [8]. Thus, often used in functional immune assays, which typically run over hours to a few days; in this context, their limited viability does not preclude utility. However, their limited lifespan and terminal differentiation restrict investigations into long-term processes, including biomineralization, developmental biology, chronic stressors, and neoplastic transformation [[9], [10], [11]]. Moreover, reliance on repeated haemocyte isolations introduces animal-to-animal variability that limits reproducibility and scalability [12,13].

To date, only two molluscan systems have been propagated beyond short-term primary culture. The *Biomphalaria glabrata* embryonic (Bge) line, derived from snail embryos, a continuous freshwater mollusc line, maintained since the 1970s and widely used to study host-parasite interactions with *Schistosoma mansoni* and invertebrate immunity [14,15]. More recently, the *Chlamys farreri* Trochophore (CfT) line, derived from Zhikong scallop trochophore larvae, was reported as the first long-term scallop culture to be established [16]. However, its designation as a continuous line will remain unresolved until key parameters, including long-term stability across subcultures are demonstrated, and the cellular composition is completely defined. Together, Bge and CfT demonstrate that molluscan cells can be maintained in extended culture, but they also highlight unresolved challenges: extremely limited taxonomic representation, a reliance on embryonic or larval sources that may not capture adult tissue physiology, and incomplete validation in terms of cell identity, passage stability, and reproducibility. Ultimately, immortalisation is not an end in itself; the value of any molluscan cell line will depend on whether it can support targeted application, provides a reproducible model for biological study, and it is genetically tractable. These challenges highlight both the technical barriers that remain and the transformative potential that could be realised if they are overcome.

Precedent from insects provide a clear success story for invertebrates: cell lines such as *Drosophila* S2 and *Spodoptera* Sf9 have become indispensable tools in virology, immunology, and recombinant protein production [17,18]. Recently, the first continuous marine invertebrate cell line was established from the sponge *Axinella donnani* [19]. Its development required customized media adapted to the osmotic and nutritional demands of sponge cells, underscoring the need to align culture conditions with the unique physiology of marine invertebrates. This achievement demonstrates that stable marine invertebrate cell lines are attainable, marking a clear path forward for similar advances in marine molluscs.

To address the limited longevity of haemocyte cultures and limits of immortalised lines for marine molluscs, explanted tissues have been employed as an alternative means of capturing and maintaining molluscan cells *in vitro*. In this context, “explants” refer to small fragments of tissue maintained under culture conditions, from which cells can shed, migrate, proliferate locally, or remain organized within the tissue. In contrast to dispersed haemocyte suspensions, explants preserve aspects of the native architecture, including cell-cell and cell-matrix interactions, which can sustain viability and functional integrity over longer intervals [6,7]. Direct evidence for extracellular matrix secretion by explanted molluscan tissues is limited. However, studies report that the addition of tissue extracts can improve culture yield and adherence, indicating that tissue-derived factors may influence the culture environment [[20], [21], [22]]. It is therefore likely that explants contribute to culture medium conditioning, which could partly alleviate the mismatch between mammalian-formulated media and the physiological requirements of marine invertebrate cells. Explants also provide access to a broader array of tissue-resident cell types, creating opportunities to study cellular diversity. Cells derived from mantle, gill, digestive gland, and gonadal tissues have most commonly been maintained *in vitro* with survival periods ranging from several days to several months [[23], [24], [25], [26]]. These systems extend culture longevity and broaden the range of cell types available; however, achieving longer-term primary culture is critical for fully characterizing molluscan cells and represents a necessary step toward developing immortalised, standardized lines.

Among bivalves, the European flat oyster, *Ostrea edulis*, is a high-priority species. It supports valuable aquaculture yet remains highly vulnerable to pathogens such as *Bonamia* spp., which continue to cause recurrent mortality events [27,28]. Beyond its commercial significance, *O. edulis* has become a flagship for large-scale restoration initiatives across Europe, where the recovery of lost oyster reefs is central to rebuilding coastal biodiversity and ecosystem services [29,30].

Recognizing this strategic importance, we developed a standardized culture method capable of supporting the long-term maintenance of multiple *O. edulis* tissues. The primary aim of this study was to establish and characterize long-term *in vitro* and *ex vivo* cultures of oyster cells and tissues. Specifically, we sought to (i) evaluate the longevity of mantle, gill, adductor muscle, visceral mass (VM), and heart explants under uniform basal culture conditions, (ii) assess tissue-level organization and activity, and (iii) characterize the diversity and behaviour of resident and shed cell populations.

Our long-term cultures of mantle, gill, VM, and whole-heart explants supported a diverse range of tissue-associated cell populations, including haemocytes, fibroblasts, myocytes, ciliated epithelial cells, and adipocytes, while revealing several unexpected phenomena during prolonged culture, including epithelial ciliation, dynamic haemocyte granule plasticity, mineral-like deposition and spontaneous microtissue formation. Together, these findings establish a tractable explant platform for investigating molluscan biology and provide a foundation for establishing more sophisticated, long-term experimental model systems.

## 2 Results

The standardized basal culture system supported the long-term maintenance of multiple adult oyster tissue explants, providing a platform for investigating tissue- and cellular-level behaviour *in vitro*. Maintenance was defined as preservation of intact structure and activity (e.g. heart contractility or cilia movement) without tissue dissociation, rather than comprehensive physiological function. Using this approach, cultures were maintained for extended periods: mantle up to 4 months, gill up to 2 months, VM for up to 8.5 months, and contracting hearts for up to 9 months. Throughout the culture period, two distinct cell populations were monitored: cells which were shed or migrated into the medium, and cells retained within the tissue explants. Shed cells included haemocytes, myocytes, fibroblast-like cells, ciliated epithelial cells, and adipocytes, many of which could be related morphologically to corresponding cell populations within the explants.

### 2.1 Ex vivo culture of intact hearts

**Fig. 1:**
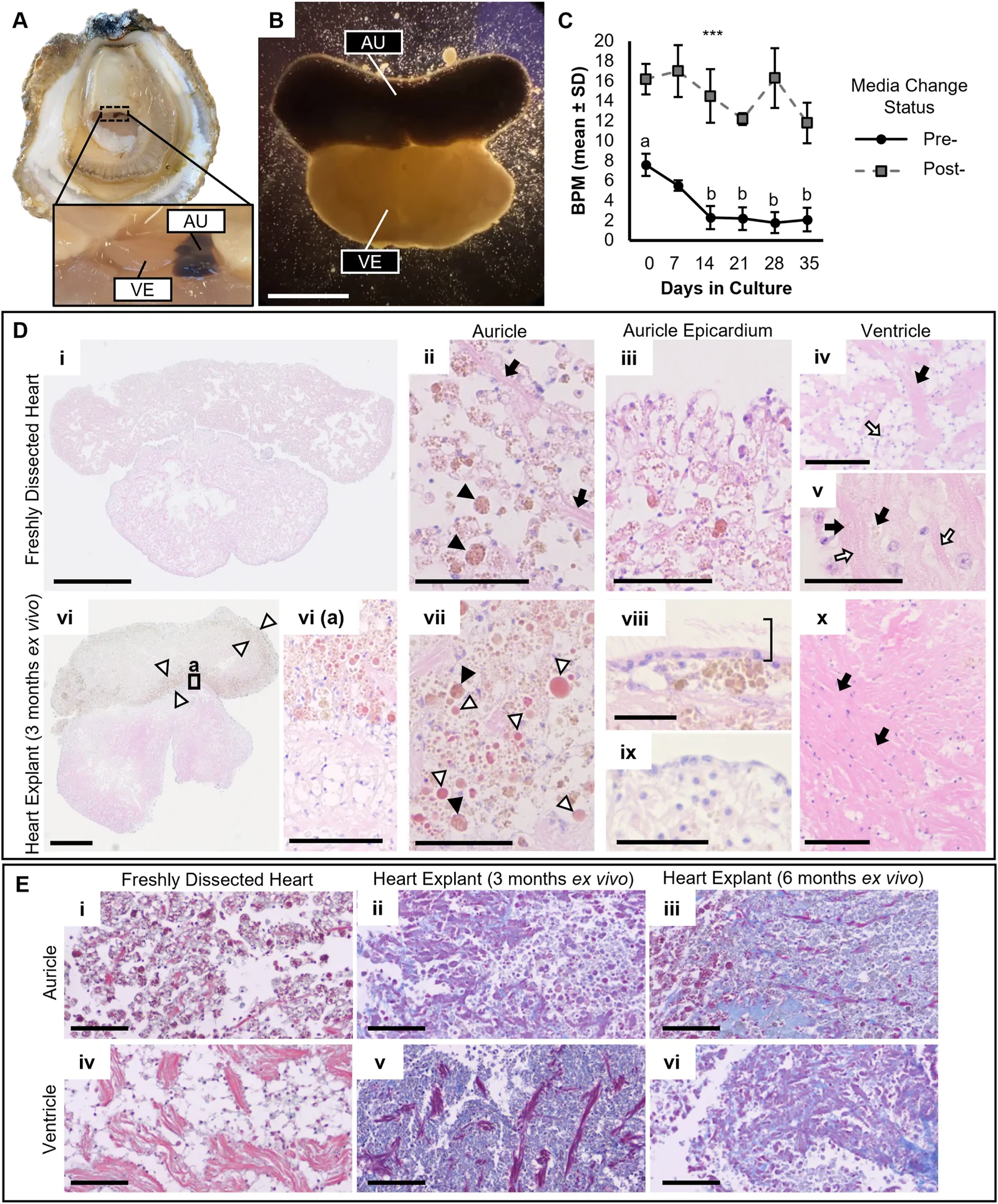
**Morphology, function and histological changes of *Ostrea edulis* hearts during long-term culture.** (A) Image of a whole oyster with the right valve removed. Inset shows an intact heart with VM and pericardium removed, exposing the auricle (AU) and ventricle (VE). (B) Image of an isolated heart explant in culture, showing distinct auricle (AU) and ventricle (VE) regions. (C) Mean ± SD beat rates measured before (pre-) and after (post-) media change at 6 timepoints in culture (n = 5 explants). Post-media change rates were consistently higher than pre-media change rates (***, p < 0.001). Letters denote significant post-hoc differences in pre-media change rates: Day 0 (a) was significantly higher than days 14 - 35 (b), p < 0.02. (D) Histological sections of freshly dissected hearts (i - v) and cultured heart explants after 3-months (vi - x) stained with H&E. Representative auricular tissue from freshly dissected hearts (ii) and cultured explants after 3 months (vii) is shown. The auricular epicardium differs between freshly dissected hearts (iii) and cultured explants (viii, ix), with cultured tissues exhibiting altered epicardial morphology, including regions of non-native ciliation. Ventricular tissue from freshly dissected hearts comprises striated cardiomyocyte bundles interspersed with fibroblast-like connective supporting tissue (iv,v) and contains lipofuscin deposits (v). After 3 months in culture, ventricular tissue exhibits denser regions of cardiomyocyte-like muscle with preserved muscle striations (x) and a reduced abundance of connective supporting tissue compared with freshly dissected hearts. Representative features are indicated: granule clusters (closed arrowheads; ii, vii), cardiomyocyte bundles (closed arrows; ii, iv, x), fibroblast-like connective tissue (open arrows, iv), lipofuscin deposits (closed arrow, v), cardiomyocyte striations (open arrow, v), region of localised concentration of hyaline globules (paired open arrowheads; vi), individual hyaline globules (open arrowheads, vii), and ciliated auricular epicardium (bracket, viii). (E) Masson's Trichrome-stained histological sections of freshly dissected hearts (i, iv) and hearts cultured for 3 months (ii, v) or 6 months (iii, vi). Representative auricular (i–iii) and ventricular (iv–vi) tissues demonstrate progressive collagen deposition (blue staining) and extracellular matrix remodelling with increasing time in culture, with more extensive collagen-rich connective tissue evident in cultured explants than in freshly dissected hearts. Scale bars: (B, D(i), D(vi)) = 2 mm; (D(ii-iii), D(vii-ix)) = 50 μm; (D(iv), D(x), D(vi)(a), E(i-vi)) = 100 μm.

Whole-heart cultures were established by transecting the veins connecting the ventricle and auricle while preserving the surrounding tissue (Fig. 1A). *In situ*, hearts displayed an elongated, stretched conformation, but in culture they adopted a more compact morphology (Fig. 1A and B). Explanted hearts retained rhythmic contractile activity for up to nine months *in vitro.* Following an initial acclimatisation period, contraction frequency remained stable over extended culture durations and reproducibly increased following media replacement.

To assess the acclimatisation time required for hearts to adjust to culture conditions, contraction rates were monitored weekly for five full weeks of culture, both before and after media replacement (S1 Table). A two-way repeated measures ANOVA showed a significant main effect of culture time, F(5,20) = 3.09, p = 0.032, and of condition (pre-vs. post-media change), F(1,4) = 144.05, p < 0.001, with no significant interaction, F(5,20) = 1.23, p = 0.332. Thus, beat rates varied across weeks and were consistently higher immediately after media replacement compared with pre-change values (Fig. 1C). Pre-media change contraction rates declined significantly over time, F(5,20) = 7.45, p < 0.001. Tukey post hoc tests indicated that day 0 rates were higher than day 14 - 35 (all p < 0.02), whereas rates between day 7 to 35 did not differ significantly (Fig. 1C). In contrast, post-media change contraction rates remained stable across the culture period, F(5,20) = 1.31, p = 0.300, suggesting that the immediate contractile response to fresh media was maintained over time. These findings demonstrate that oyster hearts retain sustained contractile activity over extended periods *in vitro*, with contraction frequency consistently increasing following media replacement, and indicate that approximately 14 days in culture is sufficient for acclimatisation. After 14 days *in vitro*, the average heart rate of explants before media replacement was 1.82 ± 2.09 bpm, whereas after media replacement it increased to 13.90 ± 5.10 bpm.

Sustained contractile activity was accompanied by the maintenance of the overall tissue architecture. In freshly dissected hearts, auricles and ventricle displayed distinct histological organisation and cellular composition (Fig. 1D(i)). The auricle was dominated by granule-containing connective tissue cells (Fig. 1D(ii)) and lined by a wavy squamous epicardium, likely reflecting relaxation and the loss of tension after dissection (Fig. 1D(iii)). The ventricle consisted predominantly of cardiomyocytes with scattered connective tissue cells (Fig. 1D(iv)).

After three months in culture, hearts were reduced in size and exhibited more densely packed cardiomyocyte tissue with preserved muscle striations, consistent with retention of architecture despite remodelling and reduction in the surrounding connective tissue (Fig. 1D(vi)). Despite these changes, clear anatomical boundaries between the auricles and ventricle were maintained (Fig. 1D(vi)(a)). No widespread necrosis or central loss of cellular architecture was observed by histology consistent with maintenance of tissue integrity during prolonged culture. The epicardium also underwent structural remodelling, with much of the auricular surface becoming lined with motile cilia (Fig. 1D(viii); S1 Video), although some regions remained non-ciliated (Fig. 1D(ix)). Ciliated epithelial regions were consistently observed between three and six months in culture, suggesting progressive epithelial remodelling under culture conditions. At the same time, the epicardial layer shifted from a squamous to a columnar-like morphology, with nuclei aligned along the basement membrane of the ciliated cells (Fig. 1D(viii)). Ventricular tissue also became increasingly compact, with a reduction in connective tissue cells separating cardiomyocytes and haemolymph sinuses (Fig. 1D(x)).

Supplementary data related to this article can be found online at https://doi.org/10.1016/j.bbrep.2026.102775

The following are the Supplementary data related to this article:

Multimedia component 3

Auricle tissue was almost entirely dominated by granule-containing connective tissue cells containing distinctive membrane-bound cytoplasmic inclusions, which occasionally aggregate into larger intracellular aggregates(Fig. 1D(vii)). These granule-containing cells were uniformly distributed throughout the auricle but absent from ventricle tissue in both fresh and cultured samples (Fig. 1D(iv), 1D(x)). Histochemical staining identified the granules as PAS-positive, indicating carbohydrate-containing material. With H&E they appeared dark pink to brown, and with Masson's Trichrome stained them dark pink, suggesting a proteinaceous component. No staining was detected with Perls' Prussian Blue, Masson's Fontana, or Toluidine Blue, excluding iron, melanin, and mast cell proteoglycans. The granules were also morphologically distinct from lipofuscin, which typically occurs as small, irregularly distributed pigment deposits (Fig. 1D(v)). In contrast, the auricular granules were larger, uniformly distributed, and present in both freshly dissected and cultured hearts, making culture- or age-related pigment accumulation unlikely. Individual intracellular granules measured an average of 1.81 ± 0.63 μm in diameter (0.82 - 3.55 μm; S2 Table), while larger aggregates consisting of multiple membrane-bound granules averaged 11.32 ± 1.90 μm (7.87 - 17.63 μm; S2 Table; Fig. 1Dg).

In addition to these intracellular granules, extracellular hyaline deposits were observed in long-term cultures. By three months, a distinct ring of hyaline globules was present along the auricular perimeter, particularly at the ventricular margins and adjacent to novel ciliated epithelial regions (Fig. 1D(vi)). These globules measured an average of 5.14 ± 1.60 μm in diameter (2.55 - 11.40 μm; S2 Table). At six months, extracellular hyaline deposits remained prominent, while the surrounding tissues exhibited increased collagen deposition, with cultured hearts appearing more fibrotic than freshly dissected or three-month explants (Fig. 1E(i-vi)).

**Fig. 2:**
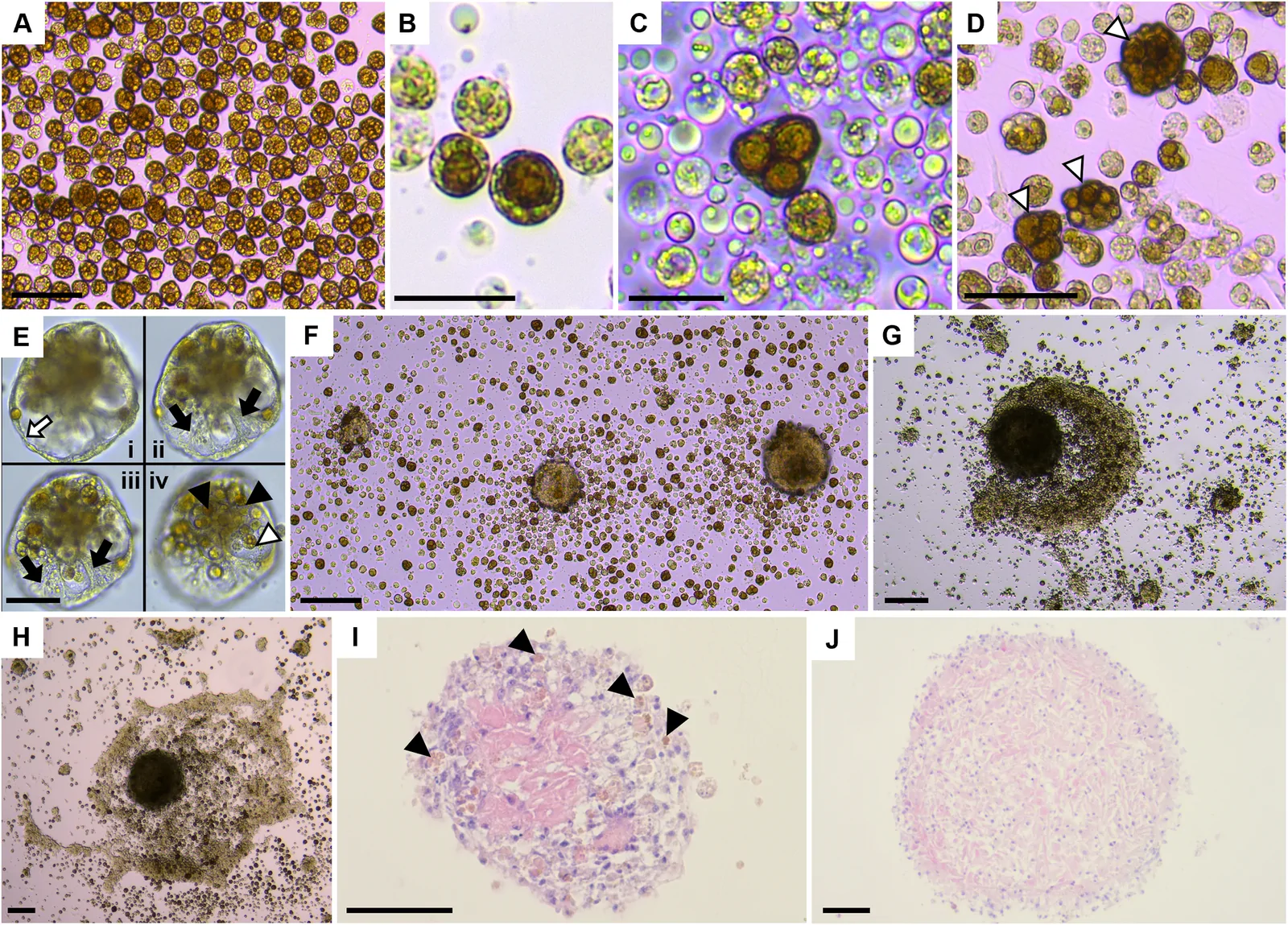
**Auricle-derived cells and emergence of contractile microtissues in cultured hearts of *Ostrea edulis*.** (A) Shed cells from auricle-only culture (ventricle removed). (B) Shed cell with concentric morphology. (C-D) Shed cell containing multiple internal bodies (white arrowheads). (E) Contracting microtissue. Sequential focal planes (i - iv) progress from the top surface (i) to the bottom adjacent to the culture plate (iv). Auricle-associated granule cells (closed arrowhead), cell containing multiple internal bodies (open arrowhead), contractile regions (closed arrow), and tissue margins (open arrow) are indicated. (F) Representative field of view showing three early microtissues, ranging from a small adherent structure (left) to larger microtissues with upward growth and defined boundaries (right). (G-H) Large contractile microtissue with concentric ring-like outgrowths. (I) H&E-stained microtissue fixed in Histogel, containing auricle-derived granule cell type. (J) H&E-stained microtissue fixed in Histogel, composed predominantly of cells consistent with cardiomyocytes. Scale bars: (A, D, E(i-iv)) = 50 μm; (B, C) = 20 μm; (F-J) = 100 μm.

When auricular tissue was cultured independently, the predominant population of shed or migrated cells in the media comprised of the granule-containing connective tissue cells observed within the parent explant (Fig. 1D(ii),1D(vii); 2A). These cells remained non-adherent throughout culture and measured 14.84 ± 1.77 μm in diameter (range: 11.26-19.14 μm; S2 Table). A subset displayed a concentric morphology, consisting of a spherical outer boundary enclosing a single densely pigmented intracellular body (Fig. 2B). In some cases, three or more such intracellular bodies were present within one cell (Fig. 2C and D). The relationship of these intracellular structures and the larger granule aggregates observed within intact auricular tissues remains unresolved.

In addition to individual cells, organized multicellular structures emerged *de novo* during prolonged heart culture, spatially distinct from the parent explants (Fig. 2E–H). Live-cell imaging captured the formation of these microtissues from small semi-adherent cell cultures, which progressively enlarged before detaching into suspension, where some exhibited spontaneous contractile activity and disperse throughout the culture dish (Fig. 2E and F; S2 Video). Larger microtissues occasionally resettled and developed concentric ring-like outgrowths (Fig. 2G and H). Unlike loosely associated suspended cell aggregates, these structures possessed well-defined boundaries and maintained a consistent multicellular organisation. Live-cell time-lapse imaging further demonstrated dynamic cellular behaviours, including progressive outward expansion of pigmented cell populations (S3 Video). The pigmented granule-containing cells shared morphological features with the granule-containing connective tissue cells observed in the auricle, suggestion an association between tissue-resident and cultured cell populations. Some microtissues expanded radially from a central core and morphologically resembled the subset of cultured granule-containing cells containing multiple intracellular bodies (S3 Video), although the cellular mechanisms underlying their formation remain unresolved. Histological analysis demonstrated that the newly formed microtissues contained cell types morphologically consistent with those observed in the parent tissue, including auricle-associated granule-containing cells and cardiomyocytes (Fig. 2I), with some structured composed predominantly of cardiomyocyte-like cells (Fig. 2J). Overtime, a subset also developed ciliated epithelial regions, consistent with the epithelial remodelling observed in long-term whole-heart explants (S2 Video).

Supplementary data related to this article can be found online at https://doi.org/10.1016/j.bbrep.2026.102775

The following are the Supplementary data related to this article:

Multimedia component 4

Multimedia component 5

### 2.2 Dynamic behaviour of haemocytes *in vitro*

**Fig. 3:**
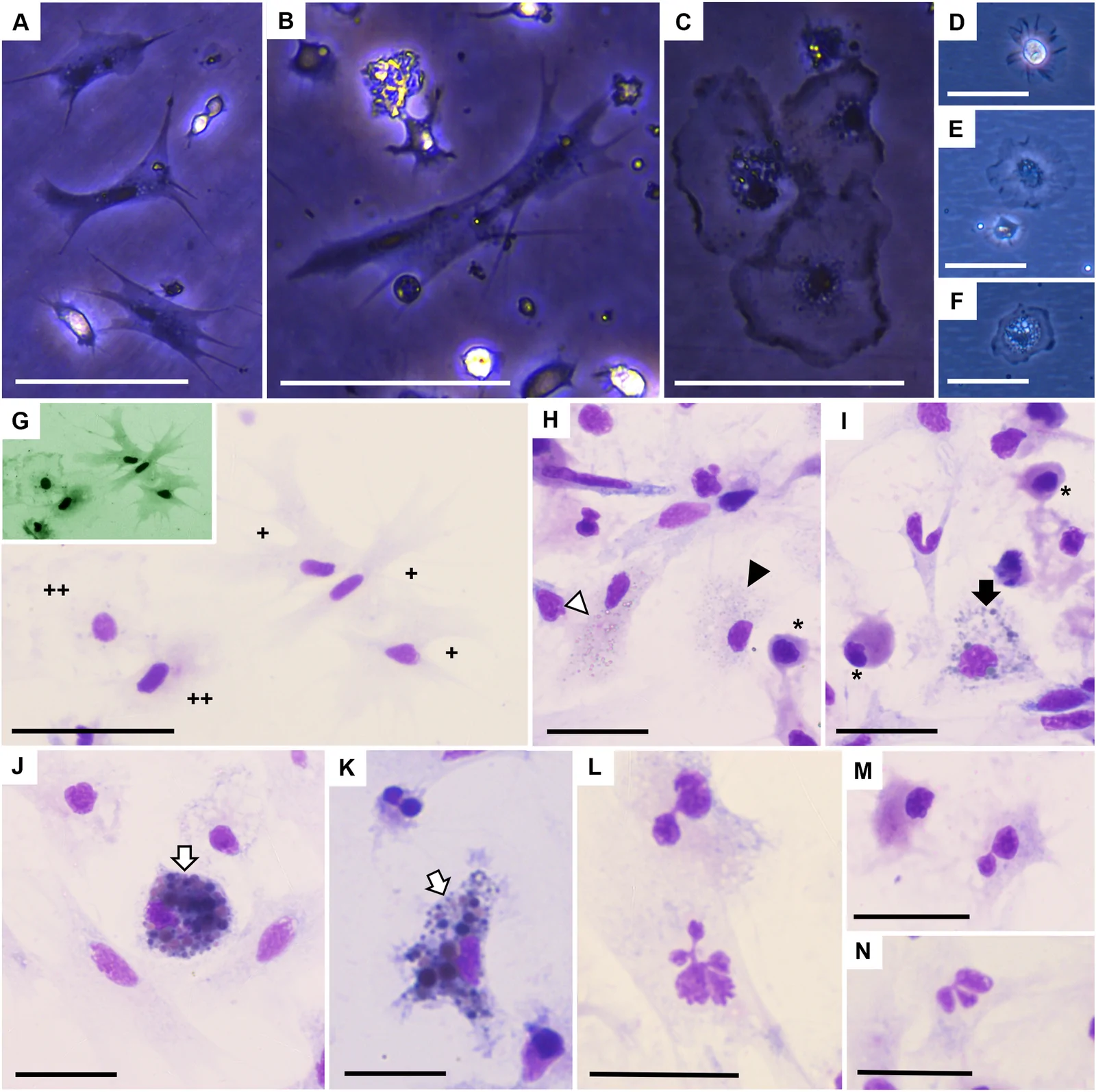
**Morphological diversity of *Ostrea edulis* haemocytes.** (A-B) Hyalinocyte-like haemocytes from fresh haemolymph, characterized by dark cytoplasm, fibroblast-like adherence, and fine filopodia; cells often overlapped upon contact. (C) Granulocyte-like haemocytes from fresh haemolymph, displaying rounded morphology, distinct nuclei, and variable cytoplasmic granulation; cells typically aggregated margin-to-margin. (D-F) Granulocyte-like haemocytes shed from explant cultures: initial adhesion (D), limited granulation (E), or extensive granulation (F). (G-K) Hemacolor-stained haemolymph preparations showing cytoplasmic heterogeneity: hyalinocyte-like haemocytes without granulation (G, single plus), degranulated granulocytes without granulation (G, double plus; inset: thallium LUT highlighting cell margins), blast-like cells without granulation (H-I, asterisk) refractive eosinophilic inclusions (H, white arrowhead), fine basophilic granules (H, black arrowhead), moderate basophilic granules (I, black arrow), and coarse basophilic granules (J-K, white arrows). (L-N) Hemacolor-stained haemolymph preparations revealed heterogeneity in polymorphonuclear cells, including bilobed (L-M), trilobed (N) and multilobed (L) forms. Scale bars: (A-G) = 50 μm; (H-N) = 20 μm.

Freshly isolated haemocytes exhibited two primary morphologies under live-cell phase contrast microscopy. The first population, morphologically consistent with hyalinocytes, exhibited uniformly dark cytoplasm, an elongated fibroblast-like morphology when adherent, and fine filopodia extending from the cell body; these cells often overlapped extensively during cell-to-cell interactions (Fig. 3A and B). The second population, morphologically consistent with granulocytes, appeared more rounded following adhesion, with clearly defined central nuclei and heterogeneous cytoplasmic granulation (Fig. 3C–F). Under phase-contrast microscopy, granules appeared as large, highly refractive inclusions, surrounded by a halo of fine dark granules concentrated in the central cytoplasm (Fig. 3C and F). These cells typically formed margin-to-margin contacts while maintaining distinct cellular boundaries (Fig. 3C). Although both morphologies were present in freshly isolated haemolymph, only the rounded granulocyte population was consistently maintained in explant cultures across all tissue types (Fig. 3D–F). Staining of fresh haemolymph further revealed additional heterogeneity, with multiple cell populations or states distinguishable by their degree of granulation and by differences in cell shape and spreading behaviour (Fig. 3G–K). Cells shed or migrated from the explant cultures closely resembled several of these haemolymph phenotypes, including cells containing small refractive inclusions (cf. Fig. 3H, white arrowhead), larger inclusions (cf. Fig. 3J and K), and rounded cells lacking visible inclusions (cf. Fig. 3G, double plus). In heart cultures, the filopodia-bearing hyalinocyte-like cells were observed only during the initial days of culture but did not persist during prolonged maintenance (cf. Fig. 3A). In addition to differences in granulation and morphology, some haemocytes displayed variation in nuclei ranging from bilobed and multilobed forms in both fresh haemolymph and cultured haemocytes (Fig. 3L–N). These nuclear morphologies occurred less frequently than the typical rounded nuclei and were not obviously restricted to a single haemocyte morphology.

Live-cell imaging demonstrated dynamic changes in haemocyte granulation during culture. Individual granular haemocytes were observed to lose their characteristic intracellular granules within 12 min, adopting an agranular phenotype, while agranular haemocytes could acquire intracellular granules within less than a minute (S4 Video). Additional modes of granulation were also observed, including membrane-associated uptake events likely demonstrating accumulation through endocytosis (S5 Video). Haemocytes were frequently motile, transitioning between fully adherent and suspended states throughout culture. The mean diameter of adherent haemocytes at their widest point was 34.96 ± 6.19 μm (range: 21.36-54.78 μm), encompassing both granular and agranular rounded forms. Diameters of suspended cells could not be reliably measured due to the presence of mixed cell types and transitional morphologies.

Supplementary data related to this article can be found online at https://doi.org/10.1016/j.bbrep.2026.102775

The following are the Supplementary data related to this article:

Multimedia component 6

Multimedia component 7

### 2.3 Cell diversity and biomineralization potential across cultured tissues

**Fig. 4:**
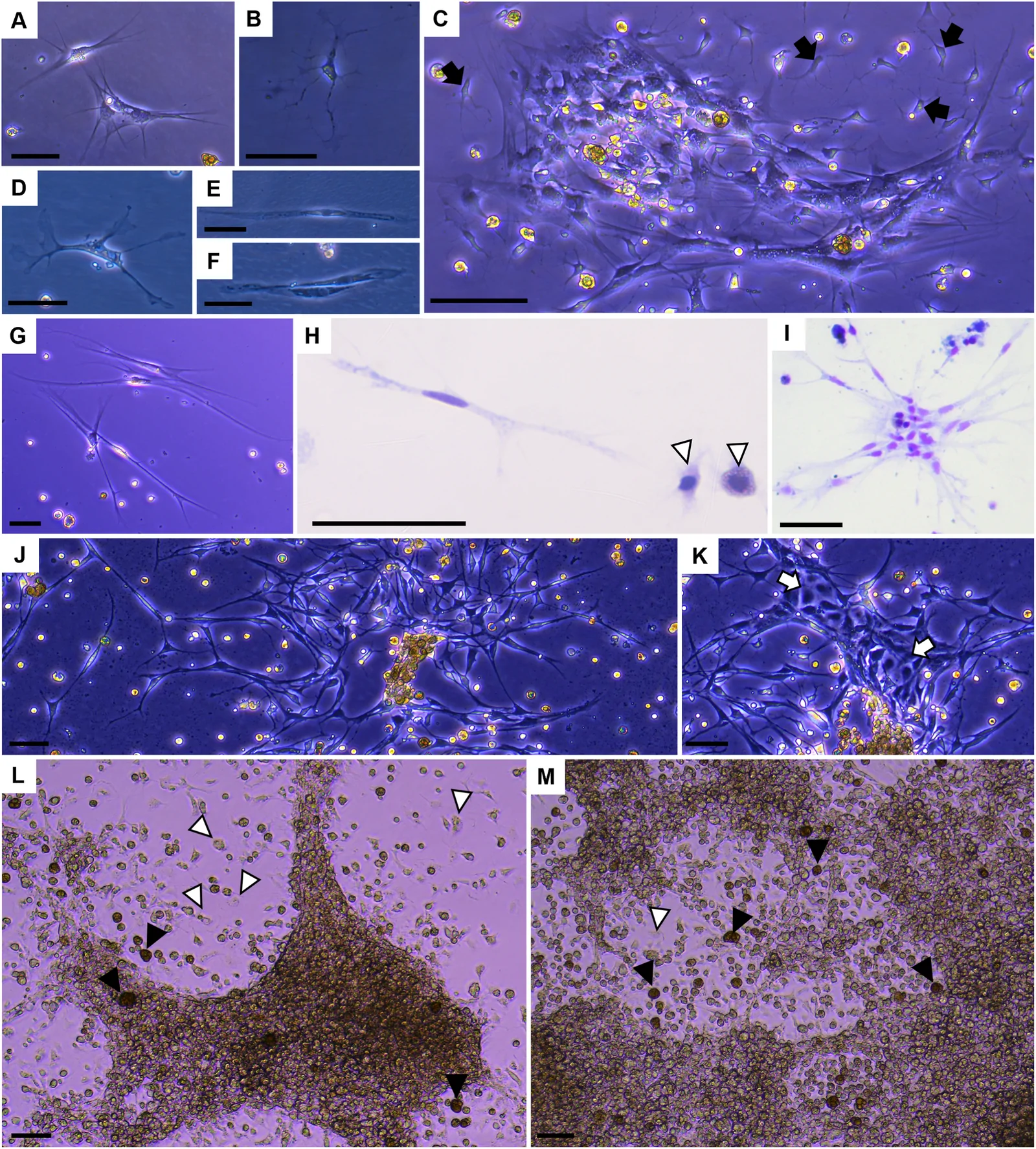
**Fibroblast-like cell diversity observed in *Ostrea edulis* explant cultures.** (A) Type I cells, exclusive to heart-derived cultures. (B) Type II cells, smaller with thin dendrite-like projections; these cells showed weak adherence and frequently detached. (C) Type I cells forming dense overlapping patches, with smaller Type II cells at the periphery (black arrows). (D) Type III cells, abundant across gill-, mantle-, and heart-derived cultures, typically stand-alone with broad, flowing projections. (E-F) Type IV cells, restricted to heart cultures, resembling cardiomyocyte-like cells with smooth, elongated morphology and alignment into stacked arrangements.(G-J) Type V cells, readily adherent and therefore more amenable to staining, showing lightly stained cytoplasm and multiple fine processes that interconnected to form multicellular aggregates and network-like structures. Haemocytes indicated by open arrowhead (K) Type V fibroblast-like networks infiltrated by additional cell types in heart cultures (white arrow). (L-M) Large, contractile multicellular clusters arising in heart cultures. Additional cell types were also present, including haemocytes (white arrowheads) and auricle-associated granular cells (black arrowheads). Scale: (A-M) = 50 μm.

Fibroblast-like cells were observed across all explant cultures and provisionally grouped into five morphological categories (Types I-V), as their precise identities could not be confirmed. The occurrence of these categories varied among explant type, with some morphologies restricted to specific tissues. Type I cells were exclusive to heart-derived cultures (Fig. 4A). These cells frequently occurred adjacent to smaller cells bearing thin dendrite-like projections, designated Type II (Fig. 4B). Type I cells maintained a distinct morphology but often formed loose associations with surrounding cells and occasionally developed dense interconnected patches (Fig. 4C). Type II cells were smaller, weakly adherent, and highly sensitive to medium replacement. They frequently detached from the substrate, establishing only transient contact with haemocytes or other fibroblast-like cells (Fig. 4C; S4 Video). Type III cells, abundant in gill-, mantle-, and heart-derived cultures, were typically observed as isolated cells with broad cytoplasmic projections (Fig. 4D). Type IV cells, only observed in heart cultures, displayed an elongated morphology resembled cardiomyocytes and occasionally aligned in parallel arrays with adjacent cells, although spontaneous contractile activity was not observed (Fig. 4E and F). Type V cells, readily adherent and therefore more amenable to Hemacolor staining, had lightly stained cytoplasm with multiple fine processes that interconnected to form multicellular aggregates (Fig. 4G–I). These aggregates frequently expanded into adherent, scaffold-like networks (Fig. 4J), which in heart cultures occasionally became infiltrated by additional cell types (Fig. 4K). Over time, some of these multicellular structed developed d into large, interconnected clusters that exhibited spontaneous contractile activity (Fig. 4L) or large monolayer-like networks (Fig. 4M). Although the precise cellular composition of these clusters could not be resolved, their organization and behaviour were distinct from smaller fibroblast-like networks, which remained non-contractile.

**Fig. 5:**
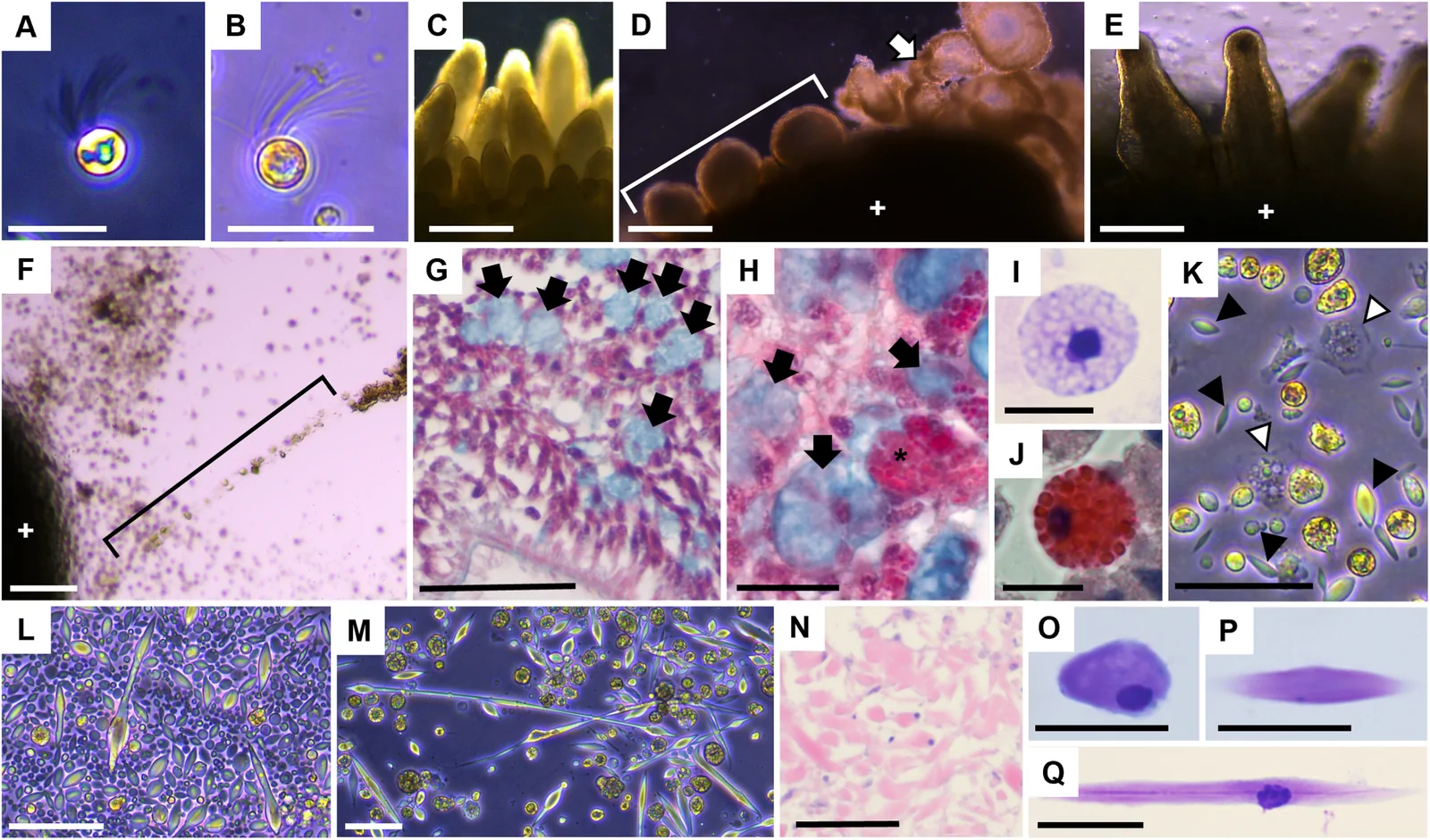
**Cellular and Tissue Features of *Ostrea edulis* Mantle and Gill Explant Cultures.** (A-B) Ciliated cells shed into culture medium. (C) Mantle lobes *in vivo*. (D-E) Mantle lobe regeneration in culture; regrowing lobes (bracket), outer dead layer shed into medium (white arrow), explant (plus). (F) Mucus in culture (bracket), explant (plus). (G) Mantle tissue stained with Masson's trichrome modified with Alcian blue; mucocytes (black arrow). (H) Gill tissue stained with Masson's trichrome modified with Alcian blue; mucocytes (black arrow) and epithelial-associated eosinophilic cell (asterisk). (I) Hemacolor staining of a mantle and gill-derived cell shed into culture medium; likely a mucocyte. (J) Hemacolor staining of an epithelial-associated eosinophilic cell shed from gill explant culture medium. (K) Unidentified round cell phenotypes. Granulocytes (white arrowhead) and myocytes (black arrowhead). (L) Smooth muscle cells (myocytes) in early culture, showing elliptical structures formed by proliferating myocytes (M) Elongated myocytes shed into medium, arising from rounded cells shown in (L). (N) Myocytes in mantle tissue from cultured explant, H&E stain. (O-P) Shed myocytes from culture, Hemacolor stain. Scale bars: (A, H, O, P) = 20 μm; (B, G, K, L, M, N, Q) = 50 μm; (C-E) = 500 μm; (F) = 100 μm; (I, J) = 10 μm.

In both mantle- and gill-derived cultures, individual ciliated cells were present in the culture medium as small, rounded cells bearing multiple motile cilia localized to one pole (Fig. 5A and B). Following decontamination, cilia were initially lost in all explants but consistently reappeared within approximately two weeks of culture. In mantle explants, decontamination resulted in formation of an outer necrotic layer and the loss of the original ciliated mantle edge (Fig. 5C–E). During prolonged culture, rounded tissue masses subsequently developed and new ciliated epithelial regions emerged at one pole of the tissue explant (Fig. 5D and E). These regenerated lobes did not replicate the full extension capacity or characteristic trilobed organisation of the native mantle edge but consistently established motile cilia along tissue margin. Weak and irregular contractions of mantle explants were occasionally observed in mantle explants but did not exhibit consistent changes following media replacement, in contract to the reproducible modulation of contractile activity observed in cultured heart tissue.

Explanted gill tissues maintained structural integrity for shorter periods, typically up to two months, although cells which were shed or migrated into the surrounding medium remained morphologically intact and active for up to one month after the parent tissue explants had disassociated. Shed populations comprised mainly haemocytes, ciliated cells, and Type III fibroblast-like cells. In addition to ciliated cells, mucous production was frequently observed during the early stages of both mantle- and gill-derived cultures and corresponded with the presence of mucocytes in the explanted tissues (Fig. 5F–H). Cytological examination of cells shed into the medium from mantle and gill explants identified a distinct eosinophilic cell type with foamy cytoplasm, morphologically consistent with mucocytes, which was only mantle- and gill-derived cultures (Fig. 5I). However, this cell type could not be reliably distinguished from other rounded suspension cells by live-cell imaging alone (Fig. 5K). A second eosinophilic cell type was often observed adjacent to mucocytes within gill tissue and was occasionally present within VM tissue and along the ventricular margin of heart explants. However, unlike mucocytes, these cells did not stain positive for mucin and were only rarely observed among cells shed into the culture medium (Fig. 5H and J).

Mantle explants gave rise to a distinct population of smooth muscle cells, myocytes (Fig. 5L and M). In early culture, these appeared as small, round cell bodies measuring 3.65 ± 1.04 μm in diameter (range: 2.04-6.95 μm; S2 Table) that subsequently elongated in opposing directions, producing elliptical structures measuring 73.82 ± 93.13 μm in length (range: 7.79-576.42 μm; S2 Table) (Fig. 5L and M). Elongated, elliptical myocytes were also shed directly into the medium from the explant, where Hemacolor and H&E staining demonstrated close morphological similarity to tissue-resident myocytes (Fig. 5N–Q). Shed myocytes did not exhibit contractile activity and progressively lost structural definition before progressively lost structural integrity over time; however, newly shed myocyte-like cells continued to appear throughout the culture period, as new myocytes continued to emerge during culture. Often, myocytes shed into the media exhibited a shrinkage of nuclei (Fig. 5P).

**Fig. 6:**
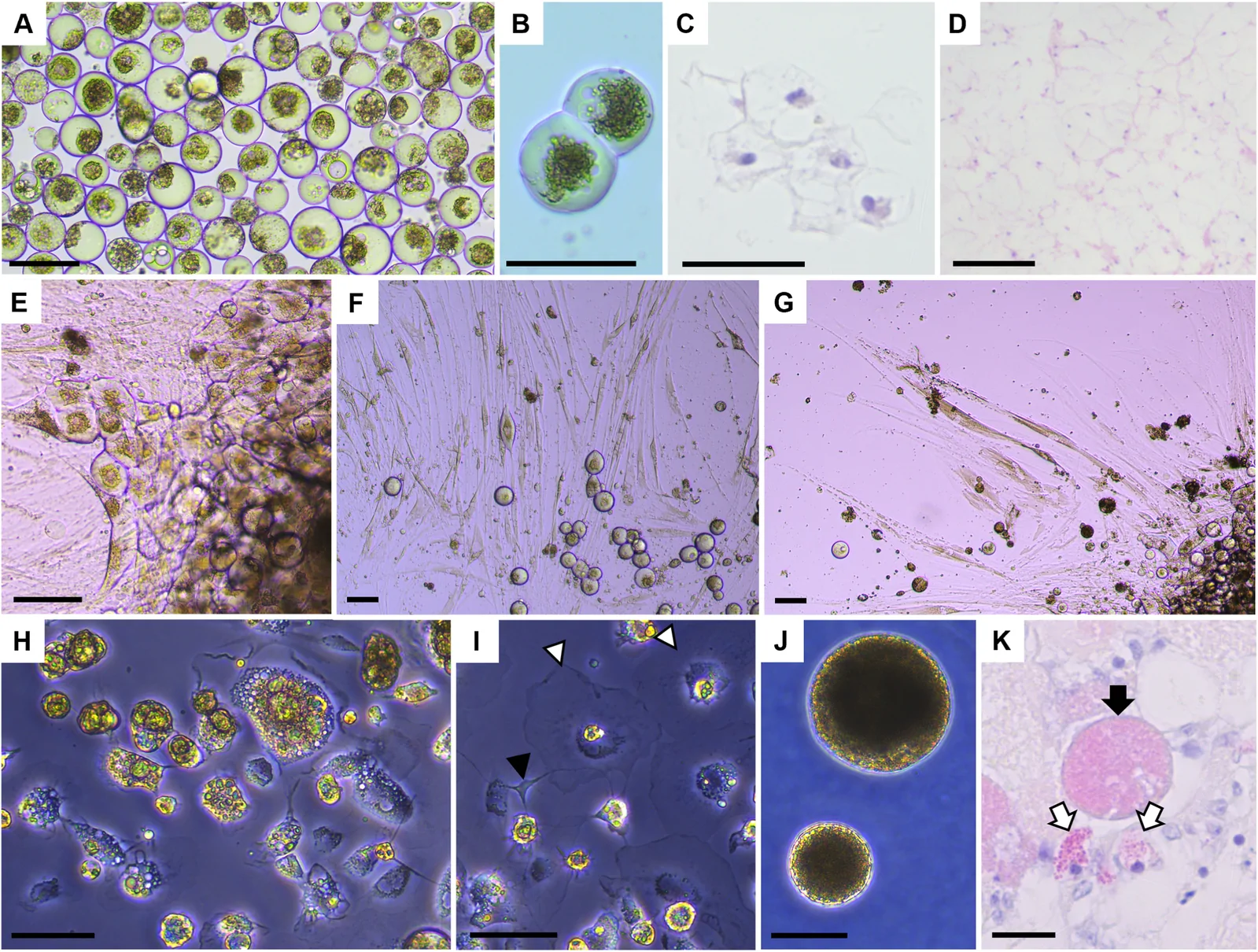
**Visceral Mass (VM) explant culture features in *Ostrea edulis*. (**A) Non-adherent adipocyte cells. (B) Adipocyte cells with polarized cytoplasm. (C) Adipocyte cells shed into culture medium, embedded in Histogel, H&E (corresponding to cells in A). (D) Adipocyte tissue within cultured explant, H&E. (E) Adherent explant showing transitional adipocyte morphology. (F-G) Monolayer of intermediate adipocyte forms and elongated cells. (H) Granular haemocytes. (I) Degranulated granular haemocytes (white arrowhead) and fibroblast type II (black arrow). (J) Large, undefined cell type specific to prolonged visceral mass cultures. (K) H&E stained 3-month cultured explant highlighting large, undefined cell type (black arrow) and eosinophilic cells (white arrow). Scale bars: (A-C, E-J) = 50 μm; (D) = 100 μm; (K) = 20 μm.

In VM explant cultures, the predominant cells shed into the medium were spherical, non-adherent adipocytes, measuring 27.07 ± 4.71 μm in diameter (range: 17.45-40.04 μm; S2 Table). These cells displayed polarized, refractile cytoplasm and sometimes occurred in closely apposed pairs (Fig. 6A and B). Their identity was supported by Histogel embedding of shed cells (corresponding to those shown in Fig. 6A; Fig. 6C) and comparison with the adipocyte population present within both fresh and cultured VM explants (Fig. 6D). Adipocytes maintained a large, rounded, non-adherent morphology for up to seven months in culture. Beyond this period, some cells adopted an elongated, adherent phenotype, anchoring the explant to the tissue culture plate (Fig. 6E–G). The appearance of these elongated cells was associated with reduced culture stability and an increased risk of complete tissue dissociation. In addition to adipocytes, the second most prevalent cell types shed from VM explants were haemocytes, present as both granular (Fig. 6H) and agranular or degranulated granulocytes (Fig. 6I), as well as fibroblast type II cells (Fig. 6I). Although the cells shed into the media largely represented haemocyte and adipocyte cells, several VM explants also shed a large, opaque cell population whose identity could not be determined using live-cell imaging or histological examination (Fig. 6J and K).

**Fig. 7:**
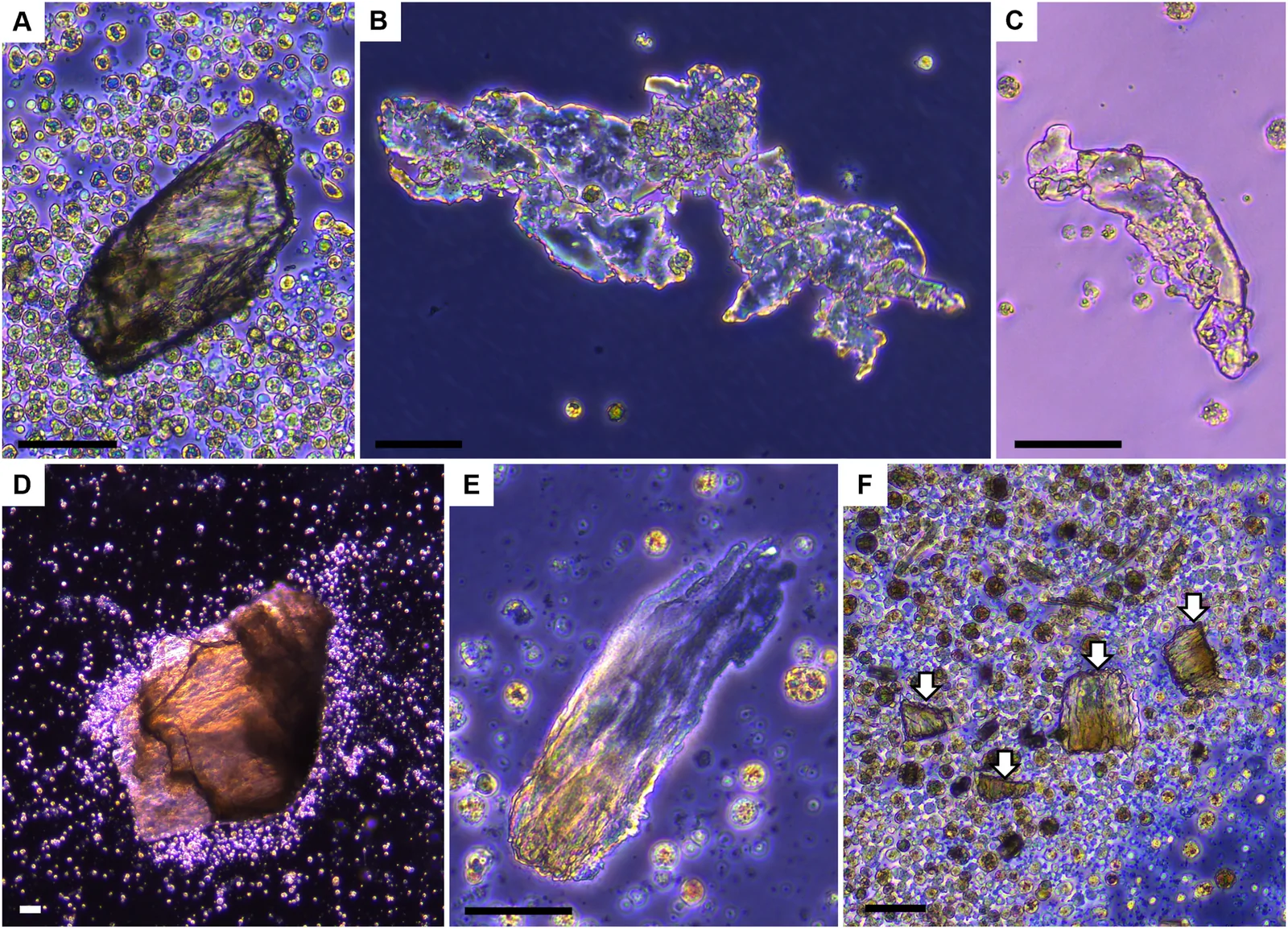
**Formation of Shell-like Structures in *Ostrea edulis* Tissue Cultures**. (A-B) Originating from heart explants. (C) Originating from mantle explant. (D) Originating from VM explant. (E-F) Originating from gill explants (black arrow). Scale bar: (A-F) = 50 μm.

Hard structured mineral-like material formed in multiple explant cultures, regardless of tissue origin (Fig. 7A–F). These shell-like structures were frequently associated with clusters of adherent cells. Their morphology varied among explant types: heart and mantle cultures produced highly refractive, nacre-like deposits (Fig. 7A–C); heart and visceral mass cultures also generated dense, opaque structures (Fig. 7A–B, 7D),while gill explants often gave rise to deposits displaying a gradient from an opaque region at one end to a progressively more translucent region at the other (Fig. 7E and F). Once formed, these structures remained stable throughout culture period and were unaffected by routine media replacement or freshwater exposure. Comparable deposits were not observed in control wells, indicating that their formation was associated with the present of explanted tissues.

### 2.4 Crosscutting culture challenges

Microbial contamination was most frequently encountered during the first 24 h following explant establishment and became uncommon once cultures stabilized. Bacterial contamination was observed most the most frequently, followed by small, unidentified ciliates, both of which occurred almost exclusively during the initial days of culture. Explants that remained free of contamination during this establishment period generally maintained long-term tissue integrity and continued to exhibit tissue-specific activities over extended culture durations. Accordingly, the first 24 h after explant preparation proved to be a useful indicator of successful long-term culture establishment.

Among successfully established cultures, approximately 75% were maintained for the longest culture durations examined in this study: mantle up to 4 months, gill up to 2 months, VM up to 8.5 months, and contracting hearts up to 9 months. Many explants were intentionally terminated at earlier timepoints for histological or imaging analyses; consequently these durations represent demonstrated longevity rather than the lifespan of every explant.

Contamination rates varied among tissue types and are presented as approximate values based on repeated independent culture attempts over a three-year period. Gill explants exhibited the highest contamination rates (approximately 30-40%), followed by mantle (20-30%),VM (15%), and heart explants (<5%). All adductor muscle explants developed persistent bacterial contamination within the first 24 h of culture, could not be maintained under the conditions used, and were, therefore, excluded from further analyses.

In addition to these technical considerations, all explant cultures consistently released abundant small non-adherent cells into the culture medium. These were morphologically distinct from granule-containing auricular cells, mucocytes, and adipocytes described elsewhere in this study. Based on their morphology and behaviour, at least a subset was consistent with non-adherent haemocytes. However, in the absence of definitive molecular markers, haemocytes could not be reliably distinguished from other small non-adherent cell populations, and the identities of these cells therefore remain unresolved.

## 3 Discussion

The establishment of long-term oyster tissue cultures represents a significant advance in the development of experimental tools for molluscan cell biology. By maintaining diverse tissue types and their associated cell populations *in vitro*, the culture system described here enables more detailed study of tissue organisation, cellular behaviour and physiological activity that has previously been difficult to achieve in molluscan systems.

A key feature of the platform developed here is the use of a single standardized basal medium to support multiple adult oyster tissues under common culture conditions. Rather than optimising media formulations for individual tissues, this approach enabled direct comparison of tissue behaviour and facilitated identification of both shared and tissue-specific cellular responses across explant types. The standardized culture system therefore established a practical foundation upon which future tissue-specific physiological studies, mechanistic investigation and media optimisation can be systematically developed.

Among the tissues examined, heart and VM explants exhibited the greatest longevity with contracting heart explants maintained for up to nine months and VM cultures for eight and a half months. Mantle explants could be maintained for up to four months, while gill explants retained tissue integrity for up to two months. Each explant type generated characteristic populations of shed or migrated cells reflecting their tissue origin: hearts produced cardiomyocyte-like cells, fibroblast-like subtypes, and haemocytes; VM cultures yielded adipocytes and haemocytes; mantle explants generated myocytes, fibroblast-like cells, and haemocytes; and gill cultures primarily released haemocytes and fibroblast-like cells. Collectively, these results demonstrate that multiple oyster tissues and cell types can be maintained long-term under common culture conditions, while exhibiting distinct patterns morphology and behaviour. The extended culture duration achieved here enabled direct observation of rhythmic contractility in hearts, epithelial ciliation, long-term persistence of adipocytes, extracellular mineral-like deposits, dynamic changes in haemocyte granulation and the emergence of *de novo* multicellular structures. Together, these observations establish *O. edulis* explants as a tractable and informative platform for future fundamental and applied studies of molluscan tissues. Importantly, the capacity of these explants to survive long-term is coupled with a measurable acclimatisation process, revealing how oyster tissues physiologically adjust to *in vitro* conditions. A key finding of this study was the acclimatisation period observed in heart explants. Isolated hearts required approximately 14 days in culture before heart rates stabilized following explantation. This likely reflects a period of adjustment which could arise from the stress of excision, adaptation to the artificial environment, or remodelling of cell-cell and cell-matrix interactions. Comparable equilibration periods have been reported in vertebrate tissue culture systems; for example, intact myocardial slices similarly require an initial period of culture before achieving stable contractile behaviour [31]. The presence of a defined adjustment window carries both biological and methodological implications: it demonstrates that oyster cardiac tissue can establish a stable long-term contractile state under *in vitro* conditions, while highlighting measurements obtained immediately following explantation are likely to reflect acute responses to tissue isolation rather than the stabilised state achieved during prolonged culture. Accordingly, functional assays are most reliable and reproducible after this initial two-week acclimatisation period.

Although equivalent functional assessment was not possible for other explant types, comparable periods of structural remodelling and adjustment were observed during early culture. In both mantle and gill explants, ciliated epithelial regions were initially lost following tissue decontamination and processing but consistently reappeared within two weeks, indicating that these tissues also undergo a reproducible period of adaptation to *in* vitro conditions. The mantle provided a clear example of tissue-specific remodelling, exhibiting both the re-emergence of ciliated epithelial regions and the continues release or migration of smooth muscle cells into the culture medium. The restoration of ciliated regions is consistent with the reported ciliogenic capacity and epithelial resilience of molluscan mantle tissue [32,33]. Similarly, the emergence of shed smooth-muscle cells parallels previous molluscan culture studies demonstrating that maintenance of myocyte morphology and contractile function is influenced by extracellular matrix and mechanical context [33,34]. Together, these observations suggest that mantle explants provide a useful complementary model for investigating epithelial remodelling, ciliogenesis and smooth muscle biology *in vitro*.

The contraction frequencies observed in cultured heart explants were lower than those reported for intact *O. edulis* under natural conditions, where heart rates typically range between 10 and 40 bpm depending on temperature and physiological state [35]. This discrepancy is not unexpected, as isolated hearts function in the absence of the haemodynamic and environmental influences present in the intact organism and would therefore be anticipated to exhibit altered basal level. Importantly, *O. edulis* is capable of marked reductions in cardiac activity under a range of physiological conditions, including valve closure, environmental stress, and periods of reduced metabolic demand, during which pronounced bradycardia and even transient cardiac arrest may occur [35]. Although the cultured explants cannot be assumed to replicate these *in vivo* physiological states, the contraction frequencies observed here fall within the know functional range of cardiac activity reported for the species.

Collectively, these observations illustrate that successful acclimatisation underlies long-term tissue stability in culture, providing the foundation for prolonged structural organisation and sustained observable physiological activity. Following an initial acclimatisation period, heart explants exhibited stable contractile activity for many months while maintaining organised tissue architecture. This unprecedented culture longevity creates opportunities to observe long-term adaptations, remodelling, and emergent cellular behaviours that are difficult to capture in conventional short-term cultures. Comparisons with other molluscan culture system highlight both parallels and distinctions. In *Magallana (*formerly *Crassostrea) gigas*, excised whole hearts continued rhythmic contractions for up to eight weeks *in vitro*, although explants gradually rounded and preservation of auricle-ventricle architecture was uncertain [26]. In *Magallana bilineata* (formerly *Crassostrea madrasensis*), small heart fragments produced migrating cells that aggregated into beating clusters surviving 25-34 days, but contractions weakened after four weeks [24]. While these cluster-based systems differ from intact heart explants, they parallel our observations that cells released from whole-heart explants can give rise to organised multicellular structures exhibiting spontaneous contractile activity during prolonged culture. Similar diversity is seen in the hard clam *Meretrix lusoria*, where fragmented heart tissues generated confluent epithelial-like monolayers after 30 days, occasionally exhibiting rhythmic contractions [25]. In the scallop *Pecten maximus*, dissociated heart tissues produced cardiomyocyte-like cells that began spontaneous rhythmic contractions after approximately 20 days, with myotube-like structures appearing at confluence [36]. These outcomes resemble the aggregated beating clusters of Balakrishnan et al. (2024) and parallel our own findings, in which Type V fibroblast-like cells developed into large multicellular scaffolding-like structures exhibiting spontaneous contractile activity, whereas Type I fibroblast-like cells formed compact but non-contractile assemblies. Together, these demonstrate that contractile activity can emerge across a range of multicellular organisations in molluscan heart cultures, including clusters, epithelial-like monolayers and long-term intact explants. *O. edulis* explant system described here extends these previous studies by combining prolonged culture longevity with maintenance of whole-organ tissue architecture over many months. Auricle-derived granule cells and the spontaneous organization of microtissues were features unique to heart cultures. This finding is particularly noteworthy, as the cellular sources driving tissue regeneration or reorganisation remains unknown in molluscs. Stem cell populations in these animals have not yet been clearly identified or localized, making our observations valuable for understanding potential mechanisms of tissue renewal *in vitro* [37]. Auricle-derived cultures were dominated by granule-containing cells, which frequently contained multiple intracellular bodies within a single cytoplasmic boundary. Time-lapse imaging revealed that these cells expanded in close association with the emergence of organised multicellular structures, while histological analysis showed that morphologically granule-containing cells were incorporated into the resulting microtissues. Whether these cells contribute directly to tissue formation or instead act as organizers initiating microtissue assembly remains unresolved.

These organised multicellular structures share several morphological with vertebrate organoids systems. However, unlike lumenized organoids, they retain centrally located cells and are composed of multiple morphologically distinct cells, making them superficially similar to multicellular spheroids while exhibiting a more complex internal organization [38]. Their emergence during prolonged culture is consistent with cellular reorganisation, although the underlying mechanisms remain unknown and contributions unrecognised progenitor populations cannot be excluded. Consequently, the identity, developmental potential, and functional role of the auricle-derived granule cells remain unresolved. Their consistent presence in both freshly isolated and long-term cultured hearts suggests that they represent a native cardiac cell population associated with the tissue remodelling observed during prolonged culture. Supporting this interpretation, Renault et al. (1995) similarly reported that the auricular “brown cells” of *O.* edulis exhibit prolonged survival and growth *in vitro*.

Terminology surrounding granular molluscan cells is inconsistent, with overlapping descriptions appearing under various names, including “pigment cells,” “brown cells,” “pore cells,” and “morula-like cells” [39,40]. Granule cells are well documented in the pericardial (Keber's) glands of several bivalves, where vesicle-rich secretory and absorptive epithelia occur. The pericardial gland itself varies among species - reported as lying on the auricular surface, lining the pericardial cavity, or forming glandular out-pockets of the pericardial wall [41]. In our histological analyses, no discrete glandular structure was detected, and to our knowledge, a species-specific description of a distinct pericardial gland *in O. edulis* has not been established. Comparable inclusion-rich cell types have also been described in other molluscs, notably the “serous” haemocytes, which share some functional features with vertebrate mast cells [40]. Nevertheless, our histochemical analyses revealed no proteoglycan staining, suggesting that the granular cells observed here do not exhibit the histochemical characteristic typically associated with vertebrate mast cells. Matozzo et al. (2025) described these “serous cells” as a rare haemocyte subtype (<1% of circulating cells) capable of degranulation and DNA trap formation for bacterial agglutination. The “brown cells” of *O. edulis* hearts described by Renault et al. (1995) may represent the same or a closely related cell population, as they similarly appeared less pigmented and less granular following prolonged culture than in freshly dissociated preparations. Consistent with this, diagnostic practices often favour ventricular over auricular imprints due to a higher abundance of serous-like cells, which may correspond to the “brown cells” described by Renault et al. (1995) and auricle-derived granular cells described here [42]. Whether these variously described populations represent a single, multifunctional cell type or reflect broader inconsistencies in molluscan cell classification remains unresolved. Collectively, these observations highlight the continuing lack of a standardised classification for granular cell populations in bivalves. At present, no defined cell type can be unequivocally matched to the predominant auricular granule-containing cells of *O. edulis*, beyond descriptive terms such as “granule,” “brown,” or “pigmented” cells. Future molecular characterisation of these cell populations will be required to determine whether various granular cell types described across molluscan taxa represent homologous cell populations or distinct functional subtypes.

Long-term maintenance of explants under *ex vivo* conditions was accompanied by structural remodelling, consistent with substantial changes in organisation and cellular behaviours during prolonged culture. Histological analyses showed that the epicardial epithelium became thicker, with cells adopting a more cuboidal morphology compared to the flattened squamous cells of freshly dissected hearts. In addition, motile cilia were observed on the epicardial epithelium covering auricular regions, a feature absent in fresh preparations. Previous studies in mollusc gills typically describe ciliary loss, damage, or functional arrest, and in some cases epithelial metaplasia [43,44]. To our knowledge, the appearance of motile cilia on an epithelial surface not normally ciliated has not been reported in molluscan tissues. Importantly, the present observations document the appearance ciliated epithelial regions during prolonged culture but do not establish whether these arise through regeneration of existing epithelial cells, differentiation of other cell populations or alternative remodelling processes. In vertebrates, ciliogenesis can be re-initiated in epithelia where cilia are part of the normal developmental program [45]. While the appearance of motile cilia on the epicardial epithelium may reflect epithelial remodelling or plasticity, alternative explanations should be considered. These include a culture-related response to prolonged stagnant conditions, a delayed form of epithelial remodelling under stress, or metaplastic changes triggered by long-term maintenance. Furthermore, the progressive increase in collagen within the *in vitro* heart culture indicates ongoing extracellular matrix remodelling. Further characterisation of fibroblast activity, collagen organisation, and extracellular matrix turnover will be required to determine whether this represents adaptive remodelling, progressive fibrosis or another culture-associated response. Another feature of long-term preparations was the appearance of hyaline globules along the auricular perimeter, often at ventricular margins and adjacent to remodelled epithelia. These structures were absent in freshly dissected hearts and consistently appeared only during prolonged culture, indicating that they are associated with long-term maintenance under *in vitro* conditions. Their proximity to ciliated regions raises the possibility of a link with localized remodelling or secretory activity, although their cellular origin and physiological role remain uncertain. Alternatively, they may reflect culture-related changes in membrane permeability, leading to altered secretion, uptake, or accumulation of material. Superficially similar structures have been described in vertebrates, including Mallory-Denk bodies in hepatocytes and eosinophilic globules in tissues of cetaceans [46,47]. Both of these vertebrate examples, however, are intracellular inclusions, whereas the oyster hyaline globules appear extracellular, and any resemblance should therefore be considered analogy rather than homology. While the composition and biological significant of these globules remain unresolved, their consistent appearance alongside epithelial remodelling and reorganisation indicates that are a reproducible feature of prolonged heart culture worthy of further investigation. In parallel, the explants released multiple morphologically distinct cell populations into the culture medium, including fibroblast-like cells, adipocytes, myocytes, haemocytes, and putative cardiomyocytes, whose organisation and behaviour could be followed over extended culture periods. The prolonged longevity of the explants also enabled directed association of many cultured cell populations with their tissue of origin. For example, adipocytes were characteristic of VM explants, whereas myocytes were a defining feature of mantle explants. Establishing these relationships between cultured cells and their source tissues provided a useful framework for refining existing terminology, particularly for mantle-derived myocytes, which exhibit a continuum of elliptical to fusiform morphologies but have frequently been grouped under the broad description of “spindle cells” in previous studies [26,48,49].

Among the diverse populations shed into the media, haemocytes were particularly notable for their dynamic behaviour in culture. While haemocytes of oysters and other bivalves are well studied *in vivo,* their activity *in vitro* revealed an unexpected degree of phenotypic plasticity. Granulocytes rapidly lost their characteristic inclusions within minutes, producing degranulated forms that resembled agranulocytes. Some of these cells later reacquired granules, suggesting a reversible transition between granular and agranular states. True agranulocytes (hyalinocytes), however, were not stably maintained under our culture conditions, suggesting that they may require distinct environmental or signalling cues for long-term persistence. Collectively, these observations demonstrate that haemocyte granulation is highly dynamic under *in vitro* conditions. Although the mechanisms responsible for granule loss and acquisition remain unresolved, these findings are consistent with sustainable phenotypic plasticity within cultured haemocyte populations and suggest that similar dynamic transitions may occur *in* vivo but remain difficult to capture using conventional static or population-level approaches [8,50,51].

Haemolymph cytology identified two principal haemocyte morphologies consistent with hyalinocytes and granulocytes. The granulocyte population exhibited marked morphological heterogeneity, spanning a continuum from agranular to highly granular cells. Within this spectrum, several distinct populations could be distinguished based on staining characteristics and cytoplasmic inclusions, including agranular granulocytes, cells containing refractive eosinophilic inclusions, and cells containing fine, moderate, or coarse basophilic granules. Whether these morphotypes represent distinct haemocyte subtypes or different physiological states within a dynamic cell population remains unresolved. Notably, a diversity of these granular haemocyte cells were maintained throughout the culture period, whereas hyalinocyte-like cells were not observed during long-term maintenance. Collectively, these findings suggest that haemocyte populations undergo substantial phenotypic change under *in* vitro conditions and highlight the importance of considering both haemocyte heterogeneity and culture-associated changes when interpreting functional or immunological studies using cultured oyster cells. Future molecular characterisation will be required to determine whether the observed continuum reflects discrete cell types, dynamic functional states, or a combination of both. More broadly, these findings accentuate the dynamic behaviours of oyster tissues during prolonged culture, revealing phenomena that extend beyond immune cell biology. One notable observation was the appearance of mineral-like extracellular deposits across multiple explant types. In bivalves, shell formation is classically associated with the periostracal groove, where specialized mantle epithelia coordinate secretion of the organic matrix and calcium carbonate deposition [52]. In the present study, however, mineral-like deposits consistently appeared during prolonged culture irrespective of tissue origin. Given the extensive washing procedures during explant preparation, the absence of comparable structures in medium-only controls, and the direct observation of their appearance during culture, these deposits are unlikely to represent residual shell fragments. Previous studies have reported mineralising activity in mantle-derived cultures [53], while haemocytes are increasingly recognised as contributing not only to immune function but also to shell repair and calcium transport [8,54,55]. More recently, stem-like “skeleton-embedding cells” with both haematopoietic and biomineralizing potential have been described in molluscs. [56]. Although the present study does not establish the composition, cellular origin or mechanisms underlying the observed mineral-like deposits, their appearance across multiple explant types highlights the utility of this long-term culture system for future investigations of mollusc biomineralization. These observations are consistent with previous evidence that mineralising activity is not necessarily restricted to mantle tissues [57] and raise the possibility that multiple oyster cell populations may contribute to extracellular mineral deposition under the appropriate conditions. Determining the composition of these deposits and identifying the responsible cell populations will be important priorities for future work.

### 3.1 Limitations and future perspectives

Collectively, these findings demonstrate that cells released from oyster explants remain biologically active during prolonged culture and exhibit diverse behaviours including dynamic changes in morphology, sustained contractility, tissue remodelling, and the emergence of multicellular structures. Observations including haemocyte granule dynamics, the diversity of fibroblast-like cells, the persistence of adipocytes, epithelial remodelling in mantle tissues, and the reproducible appearance of mineral-like deposits across explant types illustrate the broad range of cellular behaviours maintained under these culture conditions. At the same time, the identities, lineage relationships, and functional roles of many of these cell populations remain unresolved.

This highlights the interpretive limits of morphology-based classification in primary cultures. In mantle and gill explants, visible mucous material was observed, and Alcian Blue staining confirmed the presence of mucocytes in the source tissues. Secretion-like material occasionally emanated from explants *in vitro*, but whether mucocytes themselves were shed into the medium could not be determined. It remains possible that some of the rounded cells released from explants were mucocytes, illustrating the broader challenge of assigning cell identity solely on morphology. The frequent use of “rounded” as a descriptor exemplifies this challenge [26,58,59]. Rounded morphology may arise from multiple cell types, yet in the absence of lineage-specific markers such diversity collapses into a single ambiguous category, thereby masking important functional differences. Although morphology-based classification provides a useful framework for describing the diversity of cell populations maintained in culture, definitive assignment of several cultured cell types will require future molecular characterization and the development of validated lineage-specific markers for oyster tissues. Improved molecular tools will be essential to resolve the identities of cell populations and move beyond generalized morphological labels.

In addition to interpretive challenges, practical limitations remain in establishing and maintaining certain tissue types under these conditions. Although the culture conditions supported several tissue types, we were unable to establish cultures from adductor muscle. This limitation may be specific to whole adductor muscle tissue rather than muscle-derived cells in general and may be related to the compact structure of this tissue. Further work will be needed to determine whether alternative culture approaches or media composition could improve outcomes. Additionally, it remains uncertain whether similar outcomes can be replicated across other molluscs. Nonetheless, such challenges present opportunities: extending culture methods across molluscan taxa may improve outcomes for difficult tissues and reveal differences in cell types and organization. In this way, comparative approaches can both expand the technical toolkit for molluscan cell culture and deepen our understanding of invertebrate cell biology.

Together, these limitations define important priorities for future work, including molecular characterisation of cultured cell populations, lineage tracing approaches, and optimisation of tissue-specific culture conditions to fully realise the potential of this long-term explant culture platform. The establishment of long-term cultures of *O. edulis* tissues brings molluscs closer to other invertebrate model systems and provides a practical platform for both fundamental and applied research. Future transcriptomic and proteomic analyses will help define the molecular signatures of cultured cell populations, enabling the development of lineage-specific cell markers and improving cell identification. These advances could facilitate investigations into molluscan immunity, host-pathogen interactions, biomineralization, regeneration, and environmental stress responses by enabling cellular processes to be studied over timescales that were previously inaccessible. The prolonged maintenance of structurally intact and biologically active cultures also creates opportunities to further optimize culture conditions, which may ultimately facilitate the establishment of immortalised molluscan cell lines. More broadly, the long-term maintenance of intact oyster explant provides an experimental platform for investigating cellular dynamics, tissue interactions and physiological responses over timescales that have not previously been achievable in molluscan systems. As molecular and functional tools continue to develop, this platform has the potential to support advances in aquaculture, disease biology, regenerative biology, environmental physiology, and biotechnology.

## Author contributions

Conceptualization, T.P.B, T.R, and K.R.S; methodology, T.P.B., T.R., L.J.M., P.C.S., S.M., and K.R.S.; Investigation, K.R.S., and S.M.; writing – original draft, K.R.S; writing – review & editing, T.P.B., T.R., L.J.M., P.C.S., S.M., and K.R.S.; funding acquisition, T.P.B., T.R., L.J.M., P.C.S., and K.R.S.; resources, T.P.B., and K.R.S.; supervision, T.P.B., T.R., L.J.M., and P.C.S.

## Ethics declaration

This study was conducted in accordance with the ARRIVE (Animal Research: Reporting of In Vivo Experiments) guidelines as well as the following guidelines for animal welfare and/or reporting: U.K. Animals (Scientific Procedures) Act 1986. Ethics committee approval was not required. Ethical approval was not required for this study because the experimental animals were oysters (Bivalvia), which are not classified as protected animals under the UK Animals (Scientific Procedures) Act 1986.

## Resource availability

All relevant data are within the paper and its Supplementary Information files.

## Lead contact

Requests for further information and resources should be directed to and will be fulfilled by the lead contact, Kallen R. Sullivan (k.sullivan-5@sms.ed.ac.uk).

## Materials availability

This study did not generate new unique reagents.

## Declaration of competing interest

The authors declare that they have no known competing financial interests or personal relationships that could have appeared to influence the work reported in this paper.

## Figures and Tables

**Table:** Key resources table

REAGENT OR RESOURCE	SOURCE	IDENTIFIER
Biological samples		
Adult European flat oyster, *Ostrea edulis*	Commercial supplier (UK); see methods	N/A
Software and algorithms		
FIJI (ImageJ)	Schinedelin et al. (2012) https://imagej.net/software/fiji/	RRID:SCR_002285

**Fig. 8:**
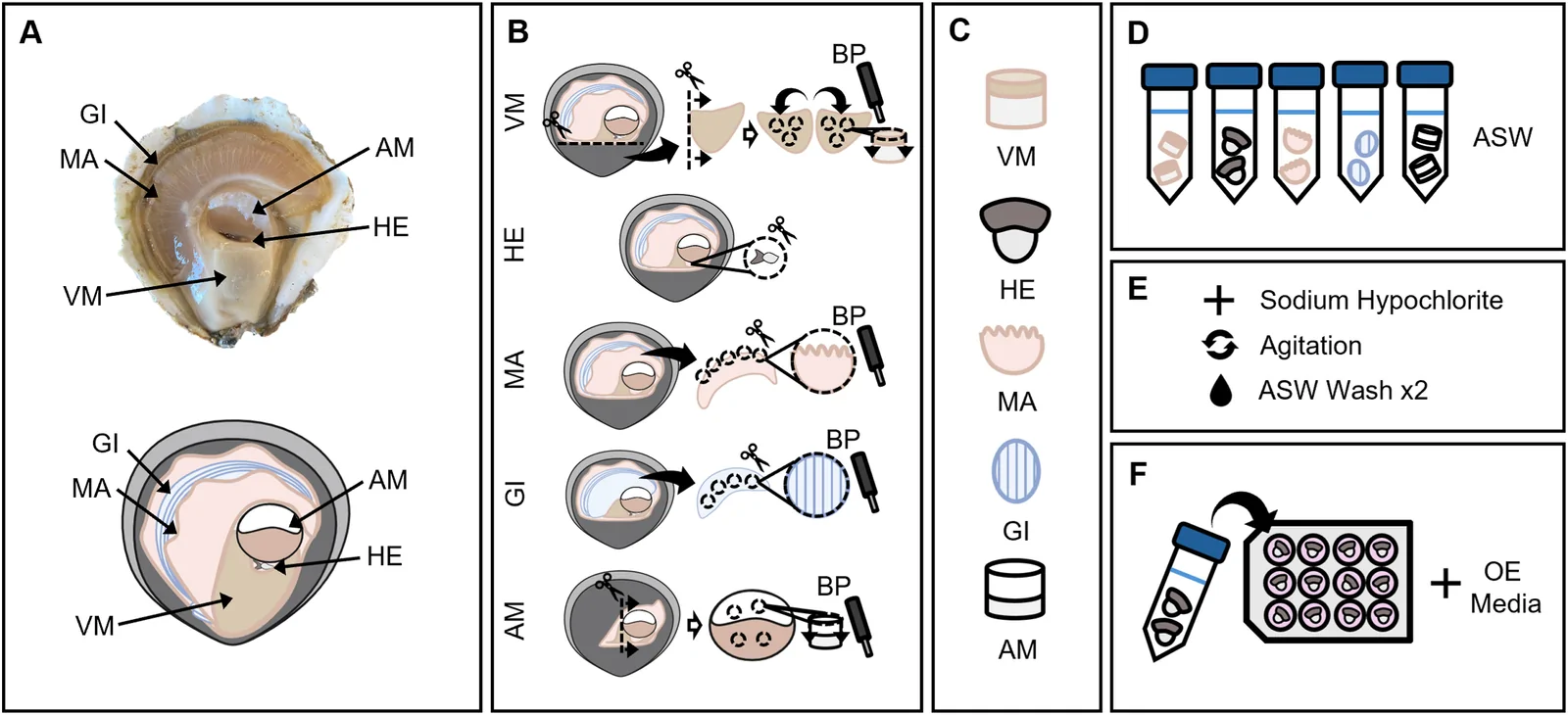
**Workflow for *Ostrea edulis* tissue explant preparation and culture.** (A) Schematic of oyster anatomy. (B) Tissue collection: visceral mass, heart, mantle, gill, and adductor muscle were dissected from whole animals and sectioned into tissue explants using spring scissors or a biopsy punch. (C) Representative icons depicting the general morphology of each tissue explant. (D) Explants were pooled by tissue type and placed into tubes containing artificial seawater. (E) Explants were surface sterilized with sodium hypochlorite, agitated, and washed twice with artificial seawater. (F) Sterilized explants were transferred into culture plates with *Ostrea edulis* (OE) media for downstream experiments. Abbreviations: AM, adductor muscle; ASW, artificial seawater; BP, biopsy punch; GI, gill; HE, heart; MA, mantle; OE, *Ostrea edulis*; VM, visceral mass.

## Data Availability

All data supporting the findings of this study are provided in the Supplementary Materials.

## Experimental model and study participant details

Adult European flat oysters (*Ostrea edulis*) were obtained from a commercial supplier in the United Kingdom and maintained as described in the Methods prior to tissue collection. Primary explant cultures were established from freshly dissected tissues. Individual oysters were not sexed because *O. edulis* is a sequential hermaphrodite and sex was not expected to influence the objectives of this study; consequently, the potential influence of sex on culture characteristics was not assessed. Ethical approval was not required because *O. edulis* is an invertebrate species not protected under the UK Animals (Scientific Procedures) Act 1986. All procedures were conducted in accordance with standard institutional procedures.

## Method details

### Oyster sourcing and storage

Adult European flat oysters (*Ostrea edulis*; small-medium class, >6.5 cm) were obtained from a commercial supplier in the United Kingdom and shipped overnight in insulated containers. For all procedures except haemolymph cytology, oysters were either dissected immediately upon arrival or stored at 4 °C and processed within two days.

For haemolymph cytology preparations, oysters were instead transferred on arrival to aerated aquaria containing artificial seawater (ASW; 35 ppt). Animals were maintained at 16 °C without feeding for one week to allow depuration, with water changed every other day before haemolymph extraction.

### Artificial seawater

ASW was prepared by dissolving Tropic Marin Classic Sea Salt (Wartenberg, Germany) in Milli-Q purified water at two concentrations: 35 g/L (35 ppt) for oyster maintenance and tissue wash/holding, or 60 g/L (60 ppt) for media preparation. All ASW solutions were sterilized by autoclaving.

### Tissue dissections and explant yield

Dissections were performed by inserting a shucking knife at the hinge, then severing the adductor muscle, and removing the right valve (Fig. 8A and B). The VM was separated along the transverse plane just below the pericardium using a scalpel to improve visibility of the heart (Fig. 8B). The whole heart was excised by removing the pericardium and tracing the margins of the auricles and ventricle with spring scissors (Fig. 8B and C). The VM was then bisected along the coronal plane to create two equal halves, shaved to a thickness of 4-6 mm, and sampled with a 6 mm^2^ biopsy punch (Fig. 8B and C). Individual layers of the mantle and gill were isolated for sampling. Mantle tissue was sampled by centring a 4 mm^2^ biopsy punch on the edge of the lobes, producing half-moon-shaped explants (Fig. 8B and C). Individual gill layers were gently stretched to ensure even tension before sampling with a biopsy punch; this yielded uniformly round explants at the time of sampling, though the natural grooves of unstretched gill tissue later caused explants to appear oval in culture (Fig. 8B and C). The adductor muscle was bisected along the coronal plane, trimmed to a thickness of 4-6 mm, and sampled with a 6 mm^2^ biopsy punch (Fig. 8B and C).

Dissections from a single adult oyster yielded one whole heart explant, approximately 6-8 digestive gland explants, 10-12 mantle explants, 12-16 gill explants, and 4-6 adductor muscle explants. All dissected tissues were pooled by tissue type and placed in 50 mL Falcon tubes containing 35 ppt ASW (Fig. 8D).

### Culture medium

*O. edulis* (OE) medium was prepared by mixing equal parts of L-15 (Leibovitz) medium (Merck) and 60 ppt ASW, resulting in an osmolality of approximately 950 mOsm/kg, as measured using an Advanced Model 3320 Micro-Osmometer (Advanced Instruments). This formulation was adapted from *Potts* et al. (2020)^26^ but adjusted to match *O. edulis* haemolymph osmolality (940 ± 9 mOsm/kg, n = 4) and to include a lower concentration of Gibco Antibiotic-Antimycotic (0.1%). The final mixture was passaged through a 0.45 μm filter before use.

### Decontamination, culture conditions and monitoring

Following dissection, tissue explants underwent a decontamination process. Samples were treated with a 0.05% (v/v) active sodium hypochlorite solution for 10 min at room temperature, with occasional agitation to improve surface sterilization. This was followed by two successive rinses in 35 ppt ASW, each lasting 10 min in a sterile tissue culture hood (Fig. 1E). After disinfection, tissue pieces were aseptically transferred to 6-, 12-, or 24-well culture plates containing the *O. edulis* culture medium with 3 mL, 2 mL or 1 mL of OE media, respectively (Fig. 1F). Plates were incubated in the dark at 20 °C. After 24 h, cultures were checked for any obvious signs of microbial contamination before being transferred to fresh wells and media.

Negative biological controls were prepared using either low-salinity (650 mOsm/kg) or high-salinity (1100 mOsm kg) media. Tissues in low-salinity media served as models to observe hypoosmotic stress, whereas those in high-salinity media were used to assess apoptotic features such as cell fragmentation and loss of adherence. Wells containing culture medium alone were maintained throughout the study as sterility controls. Explants were monitored routinely by light microscopy throughout the culture period for tissue integrity, microbial contamination, and the accumulation of shed or detached cells. Cultures exhibiting evidence of microbial contamination were immediately discarded and excluded from subsequent analyses to prevent cross-contamination.

### Live cell characterisation

Cells shed into the media were routinely examined across tissue culture types. Qualitative observations were documented throughout the culture period, focusing on cell presence, relative abundance, morphology, and behaviour.

For cytological staining, cells shed into the media (excluding non-adherent adipocytes from visceral mass explants) and fresh haemolymph were processed with the Hemacolor rapid staining system (Merck). For cells shed into culture media, 20 μL of culture medium was pipetted onto Superfrost Plus slides (Thermo Fisher Scientific), air-dried, and fixed in ice-cold 100% methanol for 7 min. For fresh haemolymph, samples were withdrawn from the adductor muscle using a sterile 18-gauge needle attached to a 1 mL syringe, placed on Superfrost Plus slides (Thermofischer), and mixed 1:1 with 35 ppt ASW to promote cell spreading. The mixture was left for 1 h at room temperature to allow haemocytes to settle, then excess fluid and debris were gently removed with ASW. Slides were air-dried and fixed in ice-cold methanol for 7 min. In both cases, fixed slides were stained according to the manufacturer's protocol, mounted with DPX medium (Sigma-Aldrich), and sealed with coverslips.

### Tissue and microtissue characterisation

Fresh, 3-month cultured, and 6-month cultured oyster tissues processed directly from the culture medium by the Histopathology Laboratory, Diagnostic Biopsy Service, The Royal (Dick) School of Veterinary Studies, University of Edinburgh, following standard operating procedures. Samples were fixed in neutral buffered formalin, embedded in Histoplast IM paraffin wax (Epredia), and sectioned at 4 μm using a HM340E microtome (Epredia). Sections were mounted on Slidemate Plus adhesion microscope slides (Epredia) and coverslipped with Pertex mounting medium (CellPath).

Microtissues recovered from culture media and isolated adipocytes were first embedded in Histogel (Epredia) prior to sectioning and were processed in parallel with paraffin-embedded tissues.

To assess tissue architecture and cellular features, standard histological stains were applied, including haematoxylin and eosin (H&E), Masson's Trichrome, Masson's Trichrome with Alcian Blue counterstain, Masson's Fontana, Perls' Prussian Blue, and Rhodanine. Staining was performed primarily using a Gemini Varistain autostainer (Thermo Fisher Scientific). When staining intensity was suboptimal, slides were restained manually to ensure consistent contrast and interpretation.

Prepared slides were scanned at ×40 magnification using a Hamamatsu NanoZoomer XR slide scanner. Digital images were archived in NDPI format and analysed with NDP.view2 software (Hamamatsu Photonics).

### Assessment of heart rate during *ex vivo* culture

The heart rates of five heart explants were monitored to assess baseline (resting) and post-stimulation (media change) activity. Explants were maintained in culture for a total of 35 days, spanning 5 full weeks of culture, and heart rate measurements were taken once weekly (day 0, 7, 14, 21, 28, 35). During each observation session, videos of beating heart explants were captured. Recordings were subsequently replayed for manual analysis, during which the number of visible contractions was counted over a 2-min interval and divided by two to calculate beats per minute (BPM). Heart explants were recorded both immediately before and immediately after media replacement.

A two-way repeated measures ANOVA was performed with Time (six observation points) and Media Replacement Status (pre-vs. post-media change) as within-subjects factors. Where significant main effects were observed, Tukey's post hoc tests were conducted to identify pairwise differences. Statistical significance was defined as *p* < 0.05.

### Image analysis and cell measurements

Microscopy was performed using multiple systems according to application. Histological slides were imaged on a Nikon ECLIPSE Ni upright microscope with 10×, 20×, and 40× air objectives and a 100× oil-immersion objective, using a ZEISS Axiocam 105 colour camera and ZEN Blue software. Microtissue time-lapse imaging was carried out on a Zeiss Axio Observer inverted microscope equipped with an environmental chamber (20 °C, no CO_2_) and a 20× objective, with images acquired on a ZEISS Axiocam 807 mono camera through ZEN Blue Pro with automated acquisition every 15 min. Manual time-lapse and live-cell phase-contrast imaging were performed on a Zeiss Axiovert 40 inverted microscope with a ZEISS Axiocam MRm camera and ZEN 2 software with manual acquisition every 30 s. Additional live-cell imaging, including microtissue and heart rate recordings, was obtained using a Leica DMi1 inverted microscope with Leica imaging software.

Following image acquisition, standard adjustments (rotation, flipping, and cropping) were applied in ImageJ (NIH, version 1.54). Digitized histological slides were analysed in NDP.view2 software, while cytology and live-cell images were measured in ImageJ. For each cell type or morphological feature, 100 measurements were collected to generate representative size distributions, except for hyaline globules, where 200 measurements were taken. Fields of view were randomly selected and all cells counted to minimize sampling bias. Results are reported as mean ± standard deviation (SD) with observed ranges.
